# Respiratory rhythm generation: triple oscillator hypothesis

**DOI:** 10.12688/f1000research.10193.1

**Published:** 2017-02-14

**Authors:** Tatiana M. Anderson, Jan-Marino Ramirez

**Affiliations:** 1 Center for Integrative Brain Research, Seattle Children’s Research Institute, Seattle, WA, USA; 2 Graduate Program for Neuroscience, University of Washington School of Medicine, Seattle, WA, USA; 3 Department of Neurological Surgery and Pediatrics, University of Washington School of Medicine, Seattle, WA, USA

**Keywords:** Respiration, breathing, rhythm generation, networks, pacemaker, preBotzinger complex, Oscillators, Postinspiration

## Abstract

Breathing is vital for survival but also interesting from the perspective of rhythm generation. This rhythmic behavior is generated within the brainstem and is thought to emerge through the interaction between independent oscillatory neuronal networks. In mammals, breathing is composed of three phases – inspiration, post-inspiration, and active expiration – and this article discusses the concept that each phase is generated by anatomically distinct rhythm-generating networks: the preBötzinger complex (preBötC), the post-inspiratory complex (PiCo), and the lateral parafacial nucleus (pF _L_), respectively. The preBötC was first discovered 25 years ago and was shown to be both necessary and sufficient for the generation of inspiration. More recently, networks have been described that are responsible for post-inspiration and active expiration. Here, we attempt to collate the current knowledge and hypotheses regarding how respiratory rhythms are generated, the role that inhibition plays, and the interactions between the medullary networks. Our considerations may have implications for rhythm generation in general.

## Introduction

Rhythms and oscillations function at the core of many brain processes ^1, 2^. For example, rhythmic spinal circuits control locomotor gait ^3, 4^, thalamic oscillations detect attentional state ^5, 6^, cerebellar rhythms are important for motor coordination ^7, 8^, and circadian rhythms entrain our biological clocks to a 24-hour cycle ^9, 10^. Compared to these circuits, respiratory neural networks in the brainstem offer a uniquely advantageous system in which to study rhythm generation because of (1) the known anatomical location of respiratory rhythm generators ^11– 15^ and (2) the ability to reduce the breathing network into various levels in preparations that retain robust and autonomous rhythmic output ^11, 15– 18^. As a result, the control of respiration can be studied from the molecular to the systems level. Mammalian respiration consists of three phases: inspiration, post-inspiration, and active expiration ^19, 20^. The networks that collectively generate the three respiratory phases are distributed bilaterally in the ventral respiratory column (VRC) of the brainstem ^21– 23^.

**Figure 1.**
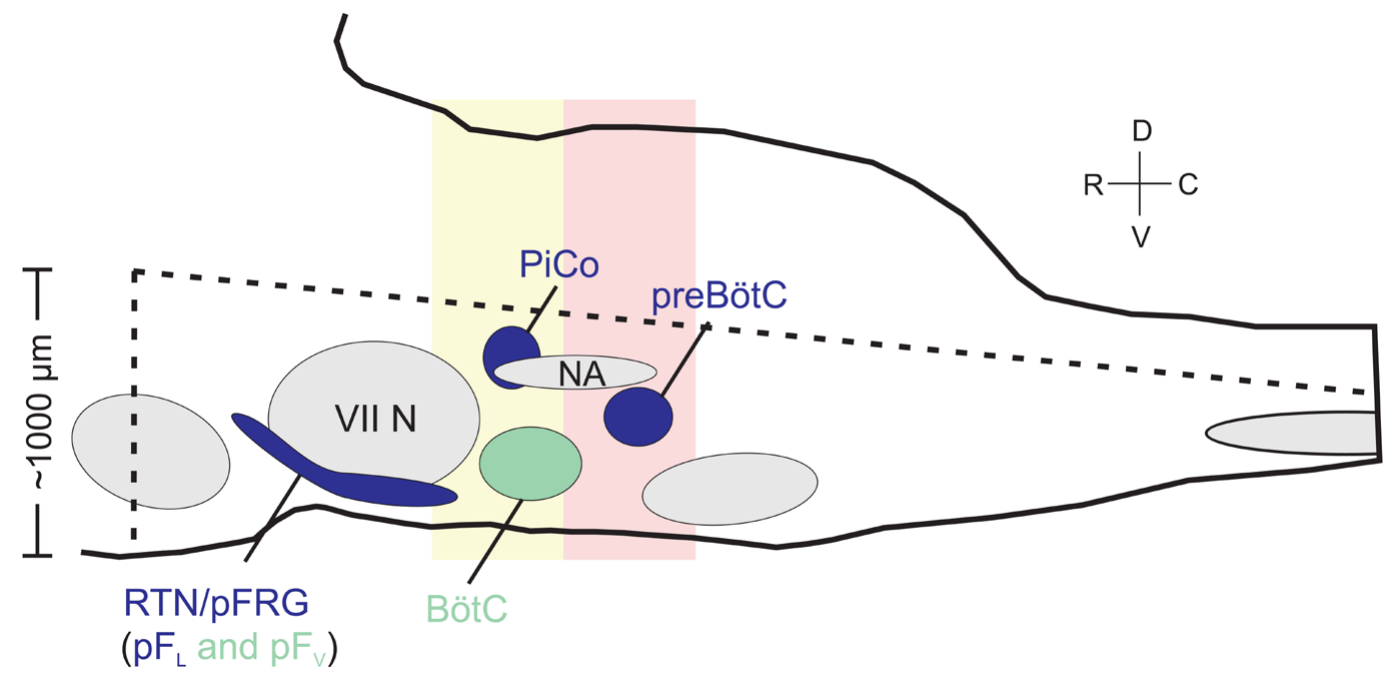
Anatomical map of oscillators in the ventral respiratory column. Schematic of the brainstem from a sagittal view illustrating the approximate anatomical locations of the three respiratory rhythm generators. Shapes in blue represent the three distinct oscillators (preBötzinger complex [preBötC], post-inspiratory complex [PiCo], and retrotrapezoid nucleus parafacial respiratory group [RTN/pFRG]) that are thought to individually control the three phases of respiration. The RTN/pFRG is further segregated into the lateral parafacial nucleus (pF _L_), which is more lateral, dorsal, and rhythmogenic, and the ventral parafacial nucleus (pF _V_), which is more medial, ventral, and not considered rhythmogenic. Shapes in gray represent motor nuclei, specifically VII N = facial nucleus and NA = nucleus ambiguus. Green represents neuronal populations that contribute to the respiratory rhythm but are not thought to be independent rhythm generators. The dotted lines indicate the approximate boundaries of the horizontal slice, while the pink and yellow boxes illustrate the approximate boundaries of transverse slices isolating preBötC and PiCo, respectively.

Within the VRC, the first described respiratory neural network, the preBötzinger complex (preBötC), is both necessary and sufficient for the generation of inspiration ^11, 24– 27^. The preBötC can singularly reconfigure to produce the inspiratory phase of eupnea (normal breathing), gasps, and sighs ^28^. The respiratory rhythm generated within the preBötC is dependent on excitatory mechanisms, and the location of the network within the ventrolateral medulla has been identified in rodents ^11, 29^, cats ^26^, and humans ^30^. Rhythm-generating, glutamatergic, and bilaterally interconnected preBötC interneurons are derived from progenitors that express the homeobox gene *Dbx1* ^25, 31^. The preBötC can be isolated in an *in vitro* transverse slice that retains fictive inspiratory bursts in phase with inspiratory hypoglossal motor output ^11^. The transverse slice is amenable to rigorous electrophysiological, histochemical, and optogenetic manipulation. Recently, two distinct rhythm generators have been described that are hypothesized to control the other two phases of respiration: the post-inspiratory complex (PiCo) for the control of post-inspiration, and the lateral parafacial nucleus (pF _L_), a subpopulation within the retrotrapezoid nucleus parafacial respiratory group (RTN/pFRG), for the control of active expiration ( Figure 1). In addition to the previously mentioned transverse *in vitro* slice ^12, 32, 33^, en-bloc ** brainstem-spinal cord ^31, 34, 35^, *in situ* ^36^, sagittal slab ^17, 37^, and, most recently, horizontal slice ^15^ preparations offer further accessibility and tractability to begin to unravel how the three phases of breathing are generated and interconnected.

## Mammalian respiratory rhythmogenesis

Decades of research have revolved around the endeavor to unmask the underlying processes controlling inspiratory rhythm generation ^11, 28, 38^. Indeed, a long-standing question in the respiratory control field queries how rhythmic, inspiratory activity in the brainstem emerges from the interaction between intrinsic cellular properties and circuit-based synaptic properties. Amid many theories, the answer remains unresolved, but it is likely that multiple rhythmogenic mechanisms exist within the functionally and molecularly heterogeneous preBötC population, and these mechanisms may vary depending on the metabolic, behavioral, and environmental conditions of the organism ^39^.

Frequently, models of neural rhythmogenesis include autonomously bursting neurons (pacemakers, or endogenous bursters) as contributors to rhythmogenesis ^40– 43^. Endogenous bursting neurons have been described in numerous rhythm-generating networks and the respiratory network is not an exception ^43, 44^. Approximately 20% of preBötC neurons can be classified as pacemakers, as defined by their tendency to burst in the absence of synaptic input at a period and burst duration similar to the duty cycle of the *in vitro* respiratory rhythm ^38, 45– 47^. Pacemaker neurons in the preBötC can be either glutamatergic ^38, 46^ or glycinergic ^48^. The “pacemaker hypothesis”, in its strictest interpretation, is the idea that excitatory pacemaker cells play an obligatory role in driving the inspiratory rhythm. It is supported by studies in which antagonists of the persistent sodium current (I _NaP_; riluzole) and the calcium-activated nonspecific cationic current (I _CAN_; flufenamic acid [FFA]), the two mechanisms underlying bursting in preBötC neurons, block fictive inspiration *in vitro* ^49^ and inspiration *in vivo* ^50^. Moreover, regions such as the preBötC and the RTN/pFRG, that are known to have rhythmogenic functions, are rich in endogenous bursters ^37^. The exact role of endogenously bursting neurons in respiratory rhythm generation is still a matter of debate ^38, 40, 43– 45, 47, 51, 52^. However, it is generally agreed that these bursting neurons do not act as simple “pacemakers” that drive the rhythm. Instead, these neurons are well integrated within the respiratory network, and synaptic and other ionic mechanisms contribute to their timing and discharge properties ^39, 40, 53, 54^.

Although cellular properties have been identified that differentiate pacemaker from non-pacemaker neurons ^55, 56^, we shouldn’t think of these in a binary manner. Instead, bursting and non-bursting lie on a continuum of firing characteristics from weak tonic firing to strong bursting ^53^, consistent with the hypothesis that preBötC neurons exhibit a continuous distribution of membrane conductances ^57– 59^. For example, I _CAN_ and I _NaP_ currents are not exclusive to endogenous burster neurons but are present on many, if not the entire population of, preBötC inspiratory neurons *in vitro* ^45, 46, 59, 60^. The “group pacemaker” theory posits that activity of tonically firing, glutamatergic preBötC neurons can percolate and increase in activity by means of positive feedback ^47, 61^. The pre-inspiratory phase occurs when the positive feedback has surpassed other network constituents and recurrent excitation leads to the initiation of a synchronized inspiratory burst ^62^.

This idea was further tested by using *in vitro* physiological data and modeling techniques to hypothesize that each individual population burst is driven by a dynamic, stochastic, and flexible assembly of preBötC neurons within a sparsely connected network ^63^. Insights into the physiology of the sparsely connected network can be performed by multi-array recordings ^64^. Using this technique, Carroll *et al*. estimated a 1% functional connectivity between preBötC neurons ^63^, a figure much lower than another study that estimated a 13% probability of one-way excitatory connectivity from dual whole-cell patch recordings of visualized, closely located preBötC neurons ^65^. Reasons for the order of magnitude discrepancy in connectivity estimates have yet to be reconciled other than obvious differences in approach and preparation.

Rhythm generation and pattern generation have been suggested to be separable phenomena ^66– 68^. Rhythm generation refers to the generation of timing signals; however, the control of the timing and coordination of muscle activity is referred to as pattern generation ^66, 69, 70^. Intracellular burst activity and motor outputs can exhibit a variety of shapes such as decrementing, augmenting, or bell-shaped ^16, 71^. Under conventional perfusion conditions *in vitro*, preBötC bursts follow a 1:1 ratio with hypoglossal motor output ^16^. However, when excitability is lowered with decreased concentrations of extracellular potassium, burst frequency decreases ^27, 72^. When a burst is expected, Feldman and colleagues instead observe “burstlets” that are small in amplitude and do not produce a motor output signal. Burstlets appear at multiples of the shortest interburst interval (i.e. are quantized) and can also be observed under specific conditions *in vivo* ^66^. The authors hypothesize that these burstlets represent pre-inspiratory activity that triggers inspiratory bursts when a certain, undefined threshold is reached.

In addition to the preBötC, two other respiratory microcircuits have been identified that function as independent oscillators controlling the other two phases of breathing: post-inspiration and active expiration ^14, 15, 73^. Under physiological conditions, expiration is a passive process and mammals largely alternate their breathing between inspiration and post-inspiration ^74^. Located rostral to the preBötC and dorsomedial to the nucleus ambiguus, the PiCo was recently identified as the putative site for the generation of post-inspiratory activity ^15^. Similar to the preBötC, PiCo rhythms are also dependent on non-NMDA, excitatory mechanisms ^15^. Thus, it is likely that the two populations employ similar rhythm-generating mechanisms. Interestingly, one study completed in goats showed that the gradual ablation of the preBötC over the course of two weeks does not result in breathing abnormalities, at least in this species, suggesting that plasticity mechanisms are able to compensate if time is allowed for brainstem networks to reconfigure ^75^. Perhaps PiCo neurons are logical candidates for assuming the preBötC’s role?

During periods of higher metabolic activity, for example during exercise, a third phase of breathing is recruited during late expiration, called active expiration, that is required to breathe air out more forcibly than under rest conditions. The active expiratory rhythm reportedly originates in the pF _L_^13, 14^. This area is defined as a conditional but independent oscillator owing to the observation that it is active only under certain conditions ^73^ but can generate rhythmic motor output from facial motor roots in the presence of an opioid agonist, DAMGO ^76^. Similar to the preBötC and PiCo, the pF _L_ is dependent on excitatory mechanisms ^35, 77^. Further studies are required to fully elucidate the rhythmogenic mechanisms of these three excitatory oscillatory networks.

## Role of inhibition

While it is generally accepted that the preBötC can burst autonomously *in vitro,* even when inhibition is blocked pharmacologically ^11^, the role of inhibition within the intact respiratory network is still debated. Originally, it was proposed that inspiration and expiration were generated by “half-centered oscillators” in which one population of neurons reciprocally inhibits the other population to generate an alternating two-phase breathing rhythm ^78^. However, these hypotheses have not been rigorously tested by specifically manipulating identified populations of neurons.

A population termed the Bötzinger complex (BötC) was discovered to contain primarily inhibitory neurons including post-inspiratory and augmenting expiratory neurons ^79– 81^. Additionally, approximately 50% of the neurons that make up the preBötC are inhibitory, mostly glycinergic, interneurons ^82^. A contemporary model posits an “inhibitory connectome” or “inhibitory ring” hypothesis in which reciprocal inhibition between the preBötC and other brainstem circuits, such as the BötC, produce the three phases of breathing ^83, 84^. The theory states that glycinergic inhibition resets the activity of inspiratory, post-inspiratory, and expiratory neurons in the ventral respiratory network ^84^. These interpretations are derived mainly from intracellular recordings *in vivo* or *in situ* paired with computational modeling (for reviews see ^18, 79, 84, 85^).

However, some aspects of this theory have been considered controversial. The inhibitory ring model would predict that blocking inhibition in the preBötC or the BötC would result in apnea, or cessation of breathing. When Feldman and colleagues tested this by pharmacologically injecting glycinergic and GABA _A_ receptor antagonists into the preBötC and BötC in vagotomized rats, they observed little to no effect on the breathing rhythm ^86^. They concluded that inhibition is not obligatory for rhythm generation but instead contributes to shaping the pattern of the rhythmic output. Of note, however, the injection of somatostatin, an inhibitory neuropeptide, into the BötC region resulted in the specific elimination of post-inspiratory vagal motor output ^87^.

These experiments were done under the assumption that the BötC was responsible for the generation of post-inspiration. However, as briefly mentioned above, it was recently discovered that the PiCo provides a necessary excitatory drive for the generation of post-inspiratory activity ^15^. The novel horizontal slice, described by Anderson *et al*., keeps the entire medullary VRC intact, and thus, using this preparation, one can simultaneously record fictive inspiratory bursts (from the preBötC) that are immediately followed by fictive post-inspiratory bursts (from the PiCo) ^15^ ( Figure 1). The PiCo rhythm persists in the absence of inhibition when the network is isolated in a transverse *in vitro* slice immediately rostral to the conventional transverse preBötC slice ^15, 88^ ( Figure 1). This is similar to the persistence of the preBötC rhythm in the absence of synaptic inhibition *in vitro* ^89– 91^. Similar to the *in vivo* experiment by Burke *et al*. ^87^, the PiCo rhythm was specifically abolished upon the application of somatostatin, with little to no change in the preBötC rhythm. Further experiments are necessary to fully elucidate the role of inhibition between respiratory rhythms *in vivo*.

## Interactions between oscillators

To truly understand how respiration is generated, it is imperative to ascertain the interactions between the different rhythm generators. While this work is far from complete, some progress has been made studying the interactions between the preBötC and the pF _L_ as well as interactions between the preBötC and the PiCo.

At embryonic day 14.5 (E14.5), before the preBötC is active, the pF _L_ is rhythmic ^35^. A day later, at E15.5, the preBötC begins to oscillate and rhythmically couples to the pF _L_. In postnatal rats, glutamatergic pF _L_ neurons provide excitatory drive to the preBötC, while the preBötC, in turn, provides inhibitory and excitatory influences on different subsets of pF _L_ neurons ^92, 93^. In the *in vivo* adult rat, the preBötC can generate an inspiratory rhythm in the absence of pF _L_ active expiratory activity ^14, 94^. However, in the converse situation, in order for the pF _L_ to be active, a second low level of activity is simultaneously required: either activity from the preBötC or increased chemosensory drive ^94^. Thus, the pF _L_ drives active expiration, but another source of excitation is required for the network to be rhythmically active.

Neurons in the pF _L_ are excitatory ^35, 76^ and do not express inhibitory biomarkers ^95, 96^. Therefore, any inhibitory action associated with pF _L_ activity must be occurring through an intermediate relay of neurons, perhaps from the preBötC ^48^. Even excitatory projections from the preBötC to the pF _L_ appear to be indirect and require an intermediate relay. Neurons in the preBötC send projections rostrally to an area adjacent to the pF _L_, the ventral parafacial nucleus, or pF _V_^97^, which has been shown to provide drive to expiration ^14, 98^, and could be functioning as the intermediate relay ^94^. While the preBötC and pF _L_ are anatomically distinct and functionally separate oscillators, the preBötC appears to be dominant, while pF _L_ activity is conditional and absent at rest.

In contrast, inspiration and post-inspiration are active at rest ^74^, suggesting that this activity may reflect the interaction between anatomically and functionally distinct oscillators, preBötC and PiCo ^15^. Horizontal slice population recordings of the preBötC and PiCo progressively synchronize when a GABA _A_ receptor antagonist is applied to the slice. This observation suggests that GABAergic connections between the preBötC and PiCo help to coordinate the timing and phasing of the respiratory rhythms.

Light stimulation of channelrhodopsin-expressing Dbx1 neurons in the preBötC simultaneously evokes inspiratory population activity in the contralateral preBötC and hyperpolarizes a post-inspiratory PiCo neuron ^15^. However, when this experiment is repeated in the absence of inhibition, light stimulation now both activates an inspiratory population burst and depolarizes the PiCo neuron. Taken together, these results suggest that, under baseline conditions, the preBötC imparts an inhibitory influence on PiCo. However, when inhibition is blocked, it unmasks a concurrent excitatory influence of preBötC onto PiCo.

This work lays the foundation for beginning to understand the dynamic interplay between the three independent rhythm generators. In particular, further studies are needed that probe the interactions between the pF _L_ and PiCo.

## Conclusion

Reduced preparations that isolate respiratory microcircuits have led to a tremendous understanding of respiratory rhythm generation. Yet, with the availability of ever-more-advanced techniques such as computational modeling, access to transgenic animals, and the possibility of working in intact, alert animals, we will further progress in the unraveling of complex mechanisms.

**Figure 2.**
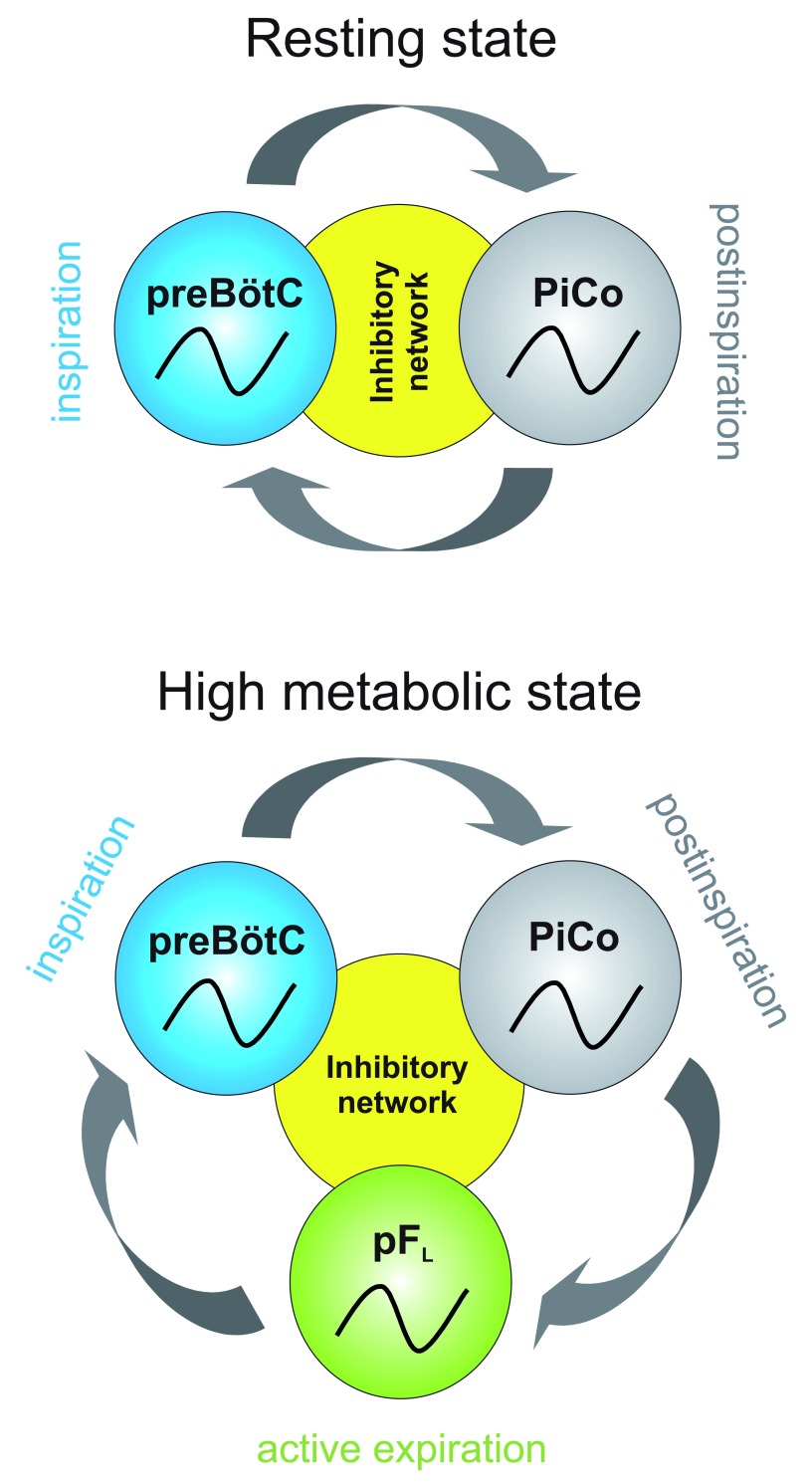
Illustration of triple-oscillator hypothesis. We propose that, at rest, the preBötzinger complex (preBötC) and post-inspiratory complex (PiCo) alternate activity to generate a two-phase rhythm, inspiration and post-inspiration. Under periods of high metabolic demand, for instance during exercise, a third oscillator is incorporated to create a three-phase rhythm. We propose that each of the three phases – inspiration, post-inspiration, and active expiration – are controlled by independent oscillators: the preBötC, PiCo, and lateral parafacial nucleus (pF _L_), respectively. We further postulate that inhibition between these networks coordinates the phasing and timing of the rhythms.

One of the most established theories for the generation of respiratory rhythms is the dual oscillator hypothesis, which posits that inspiration and expiration are generated by alternating activity between preBötC and RTN/pFRG oscillators and post-inspiration is merely a motor subcomponent of expiration ^62, 73^. We propose a triple oscillator hypothesis or that the three phases of breathing in mammals – inspiration, post-inspiration, and active expiration – are generated by anatomically distinct excitatory rhythm generators: the preBötC, PiCo, and the pF _L_, respectively ( Figure 2). It is interesting to note that three rhythm-generating networks have been hypothesized in the bullfrog ^2, 99^.

Many questions remain, however. Is there a hierarchical relationship between the three oscillators, i.e. is the preBötC the “mother of all respiratory rhythms”? Similar to the reconfiguration of the preBötC network in the generation of eupnea, gasps, and sighs, does the PiCo reconfigure to help generate post-inspiratory behaviors such as vocalization, swallowing, breath-holding, and coughing? Are the preBötC and/or PiCo networks impaired when patients with neurodegenerative disorders fail to coordinate breathing and swallowing and subsequently develop aspiration pneumonia ^100– 103^? Do homologous networks for PiCo and pF _L_ exist in humans? While substantial work remains to be accomplished, we hope that core concepts garnered from the study of the control of respiration could lead to the discovery of mechanisms that universally underlie other oscillatory networks and, ultimately, to therapies for patients with centrally derived respiratory disorders.
